# Supplementary figures and images for: Integrated Analysis of Transcriptomes and Pectinase Gene Families Reveals a Novel Pathway Mediating Tomato Fruit Malformation
Source: Int J Mol Sci. 2025 Nov 4;26(21):10739. doi: 10.3390/ijms262110739 (PMC12608487; doi:10.3390/ijms262110739)

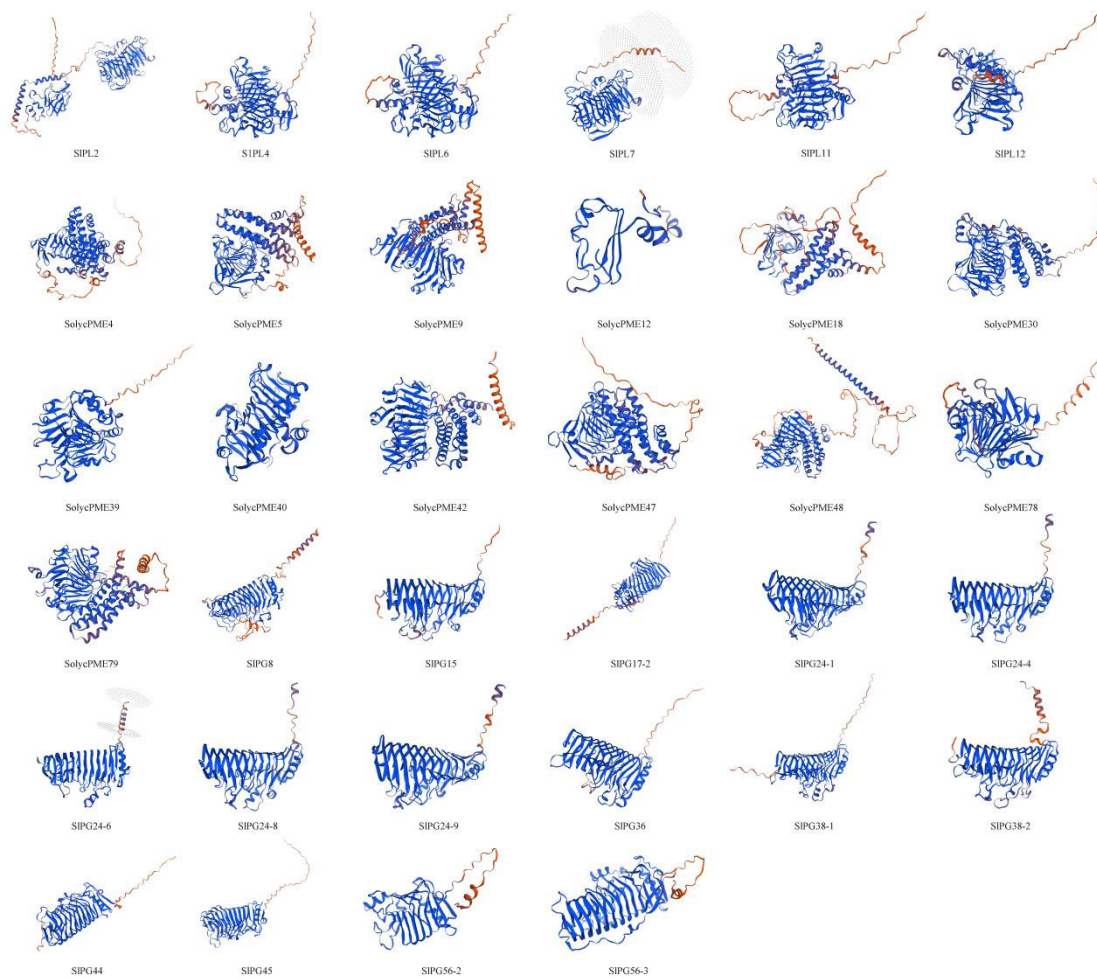

Supplement: Supplementary file 1 [file ijms-26-10739-s001.zip › Figure S1.pdf]
